# *Thiosocius teredinicola* gen. nov., sp. nov., a sulfur-oxidizing chemolithoautotrophic endosymbiont cultivated from the gills of the giant shipworm, *Kuphus polythalamius*

**DOI:** 10.1099/ijsem.0.003143

**Published:** 2018-12-12

**Authors:** Marvin A. Altamia, J. Reuben Shipway, Gisela P. Concepcion, Margo G. Haygood, Daniel L. Distel

**Affiliations:** ^1^​ Marine Science Institute, University of the Philippines, Diliman, Quezon City, Philippines; ^2^​ Department of Marine and Environmental Science, Ocean Genome Legacy Center, Northeastern University, Nahant MA 01908, USA; ^3^​ Department of Medicinal Chemistry, University of Utah, Salt Lake City, UT 84112, USA

**Keywords:** Thioautotrophic symbiont, chemolithoautotrophic symbiosis, sulfur-oxidizing chemosymbiosis, Bivalve, giant shipworm, *Kuphus polythalamia*

## Abstract

A chemolithoautotrophic sulfur-oxidizing, diazotrophic, facultatively heterotrophic, endosymbiotic bacterium, designated as strain 2141T, was isolated from the gills of the giant shipworm *Kuphus polythalamius* (Teredinidae: Bivalvia). Based on its 16S rRNA sequence, the endosymbiont falls within a clade that includes the as-yet-uncultivated thioautotrophic symbionts of a marine ciliate and hydrothermal vent gastropods, uncultivated marine sediment bacteria, and a free-living sulfur-oxidizing bacterium ODIII6, all of which belong to the Gammaproteobacteria. The endosymbiont is Gram-negative, rod-shaped and has a single polar flagellum when grown in culture. This bacterium can be grown chemolithoautotrophically on a chemically defined medium supplemented with either hydrogen sulfide, thiosulfate, tetrathionate or elemental sulfur. The closed-circular genome has a DNA G+C content of 60.1 mol% and is 4.79 Mbp in size with a large nitrogenase cluster spanning nearly 40 kbp. The diazotrophic capability was confirmed by growing the strain on chemolithoautotrophic thiosulfate-based medium without a combined source of fixed nitrogen. The bacterium is also capable of heterotrophic growth on organic acids such as acetate and propionate. The pH, temperature and salinity optima for chemolithoautotrophic growth on thiosulfate were found to be 8.5, 34 °C and 0.2 M NaCl, respectively. To our knowledge, this is the first report of pure culture of a thioautotrophic animal symbiont. The type strain of *Thiosocius teredinicola* is PMS-2141T.STBD.0c.01a^T^ (=DSM 108030^T^).

Chemoautotrophic symbiosis is a widespread phenomenon in marine environments. Numerous marine invertebrates, including certain molluscs, annelids, arthropods and nematodes, and some ciliate protozoans, associate with symbiotic bacteria, allowing them to harness energy from a wide array of compounds that are normally unavailable to animals [1]. Among the well-studied symbiotic associations are those found in marine bivalves that partner with sulfur-oxidizing chemoautotrophic (thioautotrophic) bacteria. In these bivalves, bacteria typically occur as intracellular or extracellular symbionts within specialized cells or regions of the gills. These bacteria oxidize inorganic sulfur compounds to generate energy subsequently used to fix inorganic carbon and nitrogen into biomass that can be utilized by the host [2]. Despite their widespread occurrence, thioautotrophic symbionts have eluded cultivation, thus their metabolic properties can only be inferred from their genomes and/or from data obtained using cultivation-independent techniques. In this study, we report the isolation and phenotypic characterization of the sulfur-oxidizing endosymbiont of the giant shipworm *Kuphus polythalamius*, the world’s longest bivalve [3].

Shipworms are wood-boring and wood-eating marine bivalves of the family Teredinidae [4]. Like most animals that consume wood, teredinids rely on heterotrophic (cellulolytic) bacterial symbionts to supply enzymes that aid in the digestion of wood [5–7]. Unlike more typical members of the family Teredinidae, *K. polythalamius* inhabits both wood and organic-rich marine sediments [8]. It was recently shown that this atypical species harbours intracellular thioautotrophic symbionts instead of cellulolytic bacteria in its gills [9]. Metagenomic analysis indicated that the *K. polythalamius* gill endosymbiont community is composed of several closely related thioautotrophic bacteria, one of which, strain PMS-2141T.STBD.0*c*.01*a* (hereafter referred to as strain 2141T), has been cultivated but has not yet been formally described [9]. Here, we characterize this bacterium, designating it as the type strain of the proposed new genus and species *Thiosocius teredinicola* (sulfur partner, residing in Teredinidae). The name *Thiosocius teredinicola* is effectively published in this manuscript; however, due to constraints associated with the deposition of the type strain, valid publication of this name is not possible at this time.

Isolation: strains PMS-2141T.STBD.0*c*.01*a*, PMS-2141T.STBD.0*c*.01*c*, and PMS-2146*H*.STBD.0*c*.01*a* were isolated from the gills of *K. polythalamius* as previously described [9] using STBD agar plates composed of 66 % (vol/vol) filter-sterilized natural seawater, 0.025 % (wt/vol) NH_4_Cl, 20 mM HEPES buffer, pH 8.0, 1.5 % (vol/vol) metals and mineral mix [5], 20 mM Na_2_S_2_O_3_·5H_2_O, 10 mM NaHCO_3_, 10 % (vol/vol) DMEM/Nutrient F-12 Ham (Sigma; D6421) and 1.0 % (wt/vol) Bacto agar. Colonies isolated on STBD plates under microaerophilic conditions (GasPak Campy EZ) were subsequently transferred to aerobic STB50 medium, which is similar to STBD but without the DMEM/Nutrient F-12 Ham component; and with higher concentrations of sodium thiosulfate (30 mM) and sodium bicarbonate (15 mM). In this study, three additional isolates were obtained from two specimens of *K. polythalamius* by streaking gill homogenates on STB50 solid medium incubated under normoxic growth conditions. Individual colonies were consecutively re-streaked on new STB50 plates at least three times to ensure clonal cultures. The phylogenetic identities of the resulting isolates were confirmed by sequencing 1265 bp of the 16S rRNA gene. Strain histories of *Thiosocius teredinicola* isolates are detailed in Table 1. To maintain batch-to-batch consistency during subsequent cultivations, STB50 medium was replaced with STBA, identical to STB50 except with the natural seawater component replaced with a chemically defined seawater substitute containing (l^−1^ distilled water) 23.926 g NaCl, 4.008 g Na_2_SO_4_, 0.667 g KCl, 0.196 g NaHCO_3_, 0.098 g KBr, 0.026 g H_3_BO_3_, 0.003 g NaF, 10.831 g MgCl_2_·6H_2_O, 1.518 g CaCl_2_·2H_2_O and 0.024 g SrCl_2_·6H_2_O. For all subsequent experiments, liquid cultures were grown in test tubes (16 mm×125 mm) or 250 ml Erlenmeyer flasks on a 30 °C shaker incubator (Thermo Fisher Scientific MaxQ 6000) set at 100 r.p.m. When streaked on solid media (1 % w/v Bacto agar, STBA) and incubated at 30 °C, individual colonies appeared after 3–5 days of incubation. Frozen stocks were prepared for long-term preservation by adding 40 % glycerol in distilled deionized water to a 3–5 day old STBA liquid culture at a 1 : 1 v/v ratio and freezing at −80 °C.

**Table 1.** Strain histories of *Thiosocius teredinicola* isolates.

Strain name	Source Specimen ID*	Location	Year collected	Source Tissue ID	16S rRNA accession number	Reference
PMS-2141T.STBD.0c.01a^T^	PMS-2133X *Kuphus polythalamius*	Mindanao Island, Philippines	2011	PMS-2141T (gill, posterior region)	KY643661	[9]
PMS-2141T.STBD.0c.01c	PMS-2133X *Kuphus polythalamius*	Mindanao Island, Philippines	2011	PMS-2141T (gill, posterior region)	KY643662	[9]
PMS-2146H.STBD.0c.01a	PMS-2133X *Kuphus polythalamius*	Mindanao Island, Philippines	2011	PMS-2146H (gill, anterior region)	KY643663	[9]
PMS-3412K.SNTB50.0d.02	PMS-3405S *Kuphus polythalamius*	Luzon Island, Philippines	2016	PMS-3412K (gill, anterior region)	MG097871	This study
PMS-3412K.SNTB50.0d.03	PMS-3405S *Kuphus polythalamius*	Luzon Island, Philippines	2016	PMS-3412K (gill, anterior region)	MG097872	This study
PMS-3510H.SNTB50.0a.01	PMS-3459U *Kuphus polythalamius*	Luzon Island, Philippines	2016	PMS-3510H (gill, anterior region)	MG097873	This study

*Voucher specimens are stored at the Marine Science Institute, University of the Philippines, Diliman, Quezon City, Philippines. These consist of valves, pallets, and remaining soft tissue of the *Kuphus polythalamius* specimens from which these strains were derived.

The small-subunit ribosomal (SSU) 16S rRNA gene and whole genome sequences of strain 2141T were determined previously [9]. The GenBank/EMBL/DDBJ accession numbers for the 16S rRNA gene and whole genome sequences of strain PMS-2141T.STBD.0*c*.01*a* are KY643661 and CP019936, respectively. All *T. teredinicola* isolates examined were nearly identical with respect to 16S rRNA sequence (pairwise identity >99.8 % over 1265 aligned base pairs). Phylogenetic analysis of the 16S rRNA sequences using Bayesian inference (MrBayes version 3.2.6 [10]) places *T. teredinicola* within a well-supported clade that contains the as-yet-uncultivated thioautotrophic ectosymbiont of marine ciliates provisionally named as *Candidatus* Thiobios zoothamnicoli [11], an esophageal endosymbiont of hydrothermal vent gastropods [12, 13], a variety of uncultivated Gammaproteobacteria from marine sediments, and a single cultivated, but as yet undescribed, free-living thioautotrophic bacterium, strain ODIII6, isolated from a shallow-water hydrothermal vent [14] (Figs 1 and S1, available in the online version of this article). This clade falls within a larger clade of Gammaproteobacteria previously shown to contain many thioautotrophic bacteria, including cultivated and uncultivated free-living bacteria and uncultivated symbionts (Fig. S1) [1].

**Fig. 1.**
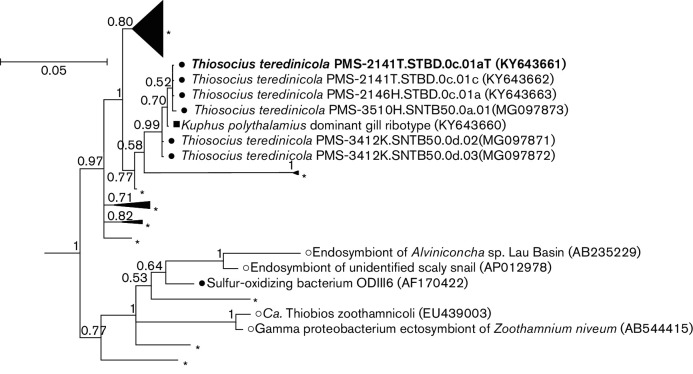
Phylogeny of *Thiosocius teredinicola* strains and other related symbionts and free-living bacteria based on Bayesian inference analysis of 16S rRNA sequences. The tree presented is an excerpt of a Bayesian tree (Fig. S1) reconstructed using 1172 nucleotide positions employing GTR+I+Γ as the substitution model in MrBayes version 3.2.6. Chain length was set to 4 million, subsampling every 2000 generations and discarding the first 20 % of the analytical results as burn-in. Posterior probability values are indicated for each node. The scale bar represents nucleotide substitution rate per site. Closed circles, bacterial isolates; open circles, uncultivated symbionts; asterisks, environmental clones; closed square, sequence recovered from *K. polythalamius* gill metagenome [9].

The closed-circular genome of strain 2141T comprised 4 790 451 bp and had a G+C content of 60.1 % and 4498 predicted protein-coding genes. This is considerably larger than the genome of the uncultivated esophageal endosymbiont of the scaly snail (gastropod), the only member of this clade with available genome data, which contains 2.59 Mbp and a G+C content of 65.1 % [12]. Consistent with this, strain 2141T did not show genome characteristics commonly observed among obligate intracellular symbionts, such as small genome size, low % G+C content and accumulation of deleterious mutations that result in pseudogenization of genes crucial for free-living existence [15, 16]. These observations, combined with the fact that it has no specific host-dependent nutritional or growth requirements, suggest that strain 2141T is likely a facultative intracellular symbiont and is possibly acquired by the host from a free-living pool in the environment, as has been proposed for the thioautotrophic symbionts of hydrothermal vent tubeworms [17, 18] and bathymodiolin mussels [19]. Strain 2141T and the scaly snail symbiont, its closest relative with available genomic sequence data (as yet uncultivated), share 93.8 % sequence identity in their 16S rRNA genes and 77 % average nucleotide identity (ANI) across their sequenced genomes. Similarly, strain 2141T shares 90.5 % identity in 16S rRNA genes and 71 % ANI with *Sedimenticola thiotaurini* SIP-G1 [20], the closest member of a validly named species (see Fig. S1). These values indicate a substantially lower degree of sequence identity than values (98.65 and 95 %, respectively) frequently cited to justify the inclusion of strains within a single bacterial species [21, 22]. The percentage of conserved proteins determined between the genomes of *T. teredinicola* and *Sedimenticola thiotaurini* SIP-G1 is 49.5 %, a degree of identity less than the lower boundary of 50 % recently proposed to justify inclusion of species within bacterial genera [23].

Consistent with culture-based observations, analysis of the genome suggests that strain 2141T is capable of oxidizing a wide variety of reduced sulfur compounds and can fix carbon dioxide autotrophically using a carboxysome-associated ribulose-1,5-bisphosphate carboxylase/oxygenase (RuBisCO). Strain 2141T also has the ability to fix atmospheric dinitrogen due to the presence of a predicted large *nif* gene cluster containing molybdenum and iron-acquisition genes, electron transport complex genes *rnfABCDGE*, core nitrogenase *nifHDKT* genes in a single operon and accessory genes *nifQBALENXUSVPWZ.* In addition, genome analysis predicts a complete TCA cycle and glyoxylate bypass pathway, implying that strain 2141T can grow heterotrophically using various organic compounds such as acetate and propionate. A more detailed analysis of the genome will be published elsewhere.

When grown autotrophically on STBA medium, strain 2141T appeared as unattached individual Gram-negative rods, that are on an average, 1.7 µm long and 0.5 µm wide. Transmission electron micrographs indicate the presence of polyhedral bodies suggestive of carboxysomes and electron-lucent intracellular objects similar in appearance to sulfur storage globules observed in many thioautotrophic bacteria (Fig. 2a). Energy-dispersive x-ray analysis, performed as previously described [9], demonstrate that these globules are highly enriched in sulfur as compared to surrounding cytoplasm (Fig. S2). Negative-staining of cultivated 2141*T* cells with 1.5 % phosphotungstic acid revealed the presence of a single-polar flagellum per cell (Fig. 2b). Colonies of *T. teredinicola* were circular, with smooth edges, uniform size and had chalky white or light yellow coloration, likely due to the accumulation of intracellular sulfur globules. Cellular fatty acid methyl ester (FAME) profiling identified C_16 : 1_ ω7*c* and/or C_16 : 1_ ω6*c* (summed feature 3 in Table S1) (40.1 %), palmitic acid (C_16 : 0_) (30.4 %), and (C_18 : 1_ ω7*c*) (summed feature 8 in Table S1) (11.9 %) as the major lipid components of strain 2141T (Table S1). The FAME profile of strain 2141T did not show any significant matches to known strains maintained in the Sherlock fatty acid reference library maintained by midi.

**Fig. 2.**
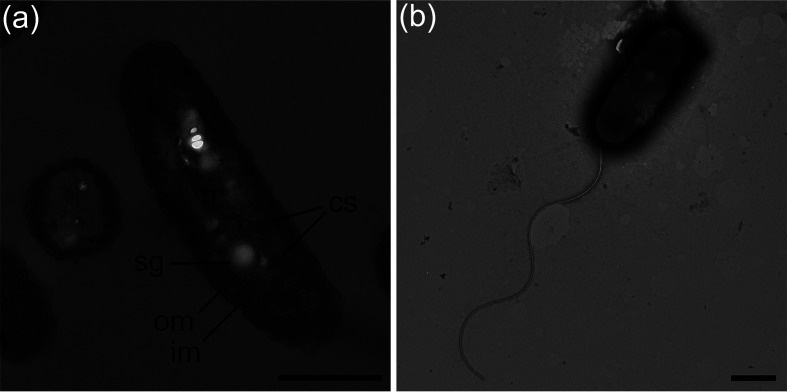
Transmission electron micrographs of *Thiosocius teredinicola* strain 2141T. (a) Longitudinal section showing nucleoid (n), sulfur globule (sg), carboxysomes (cs), outer membrane (om), and inner membrane (im). (b) Negatively-stained cell showing the presence of a single polar flagellum. Cells were grown chemolithoautotrophically in STBA liquid culture medium. Bars, 500 nm.

To evaluate the requirements of strain 2141T for autotrophic growth, inorganic carbon (bicarbonate), reduced sulfur (thiosulfate) and combined nitrogen (ammonium chloride) sources were systematically removed from the STBA liquid medium. As expected, no growth was observed in the absence of thiosulfate. However, growth was observed without added bicarbonate and/or ammonium chloride, indicating that dissolved atmospheric carbon dioxide and nitrogen are sufficient to support growth. Growth in the absence of added bicarbonate is consistent with the observation of carboxysome-like particles in the cell cytoplasm (Fig. 2) and genes encoding carboxysome-associated shell proteins, RuBisCO and carbonic anhydrase in the genome. Carboxysomes are bacterial microcompartments commonly found in free-living chemoautotrophs and cyanobacteria [24] where they function to concentrate carbon dioxide for carbon fixation. To date, carboxysome-like structures have been observed in few symbiotic sulfur-oxidizing bacteria, including the sulfur-oxidizing ectosymbionts of the nematode *Catanema* [25], the endosymbionts of the bivalve *Solemya occidentalis* [26] and the ciliate protozoan *Zoothamnium niveum* [27]; however, these observations have not been corroborated with physiological, biochemical or molecular evidence. RuBisCo activity consistent with chemoautrophy has previously been reported for cultures of 2141T [9]. Similarly, growth without an added source of combined nitrogen is consistent with the presence of a predicted *nif* gene cluster encoding all essential genes for biological nitrogen fixation in strain 2141T. Nitrogen fixation by animal endosymbionts was first demonstrated in the shipworm *Lyrodus pedicellatus* [28]. It has recently been reported that several sulfur-oxidizing symbionts are also capable of nitrogen-fixation [29, 30], thus diazotrophy among bacteria-animal symbioses might be more widespread than previously thought.

Next, we tested whether sulfur compounds other than thiosulfate can support thioautotrophic growth of strain 2141T. To test if strain 2141T can utilize sulfide, soft agar stabs consisting of 4.0 ml agar plug of SBA medium (identical to STBA except that thiosulfate was omitted) with 5.0 mM freshly prepared Na_2_S·9H_2_O (1 % w/v Bacto agar) overlaid with 8.0 ml soft SBA agar without sulfide (0.2 % w/v Bacto agar) were prepared. The tubes were capped tightly and incubated at 4 °C for 2 days to allow the oxygen-sulfide gradient to form. The soft agar tubes were then inoculated by stabbing the agar with liquid cultures grown autotrophically on STBA, or heterotrophically on sodium propionate. After 3 days, a turbid layer of cells formed below the agar surface in the inoculated tube, characteristic of growth on sulfide. No similar layer was observed in inoculated soft agar tubes without added sulfide or in uninoculated controls, although, as expected, a faint layer of abiotically precipitated sulfur was present in the uninoculated, sulfide-containing tube. Growth was also observed on SBA liquid culture containing tetrathionate (5 and 10 mM), or elemental sulfur (1 % w/v) in place of thiosulfate, but not sulfite (1, 2.5, and 5 mM) or sulfate (0.1 % w/v). These data are consistent with the bioinformatic predictions based on whole genome sequence data for this strain. Absence of growth on sulfate, the terminally oxidized form of sulfur, is consistent with the proposed role of reduced sulfur compounds as substrates for oxidative phosphorylation.

In addition to autotrophic growth, strain 2141T was also observed to grow heterotrophically on S medium (identical to STBA except that thiosulfate and bicarbonate were omitted) supplemented with malate, succinate, propionate, acetate, pyruvate or fumarate at 0.1 % w/v, or glycerol or ethanol at 0.5 % w/v, but not citrate, malonate or lactate at concentrations of 0.1 % w/v, respectively. This result suggests the possibility that strain 2141T may be capable of utilizing organic acids, alcohols or other metabolic waste products produced by its host. A metaproteomic study of *Olavius algarvensis*, a gutless and mouthless marine nematode, inferred that its thioautotrophic γ1 extracellular endosymbiont can grow heterotrophically using metabolic wastes such as organic acids that are generated by the host during periods of hypoxia. This was proposed to improve the overall metabolic efficiency of the symbiosis by recycling the waste generated by the host and using it as an alternative energy source for the symbiont [31]. It is possible that a similar strategy may be employed in the *K. polythalamius–T. teredinicola* symbiosis.

In addition to organic acids and alcohols, we also tested to determine if strain 2141T can utilize free l-amino acids. We tested for growth in S medium supplemented with each of following soluble amino acids individually: alanine, arginine, asparagine, cysteine, glutamate, glutamine, glycine, histidine, isoleucine, methionine, phenylalanine, proline, serine, threonine and valine, all at 0.1 % w/v final concentration. Of these, only glutamine and glutamate supported growth of strain 2141T, albeit only weakly.

Previously examined endosymbionts of shipworms are capable of growth on a variety of wood-associated sugars and polysaccharides. We tested strain 2141T for the ability to grow on an assortment of wood-associated carbohydrates including cellobiose, d-xylose, d-glucose, d-fructose, sucrose, d-galactose, d-mannose, d-mannitol, d-arabinose and carboxymethylcellulose, all at 0.5 % w/v on S medium. Of these, only d-fructose supported growth.

A gene cluster similar to the methylamine utilization cluster present in many methylotrophic organisms was identified in the genome of strain 2141T. However, under the conditions tested [S medium supplemented with methylamine·HCl (0.1 % w/v) under normoxic growth conditions], no growth of strain 2141T was observed. Various other C1-compounds (methanol, formate, formaldehyde, formamide and methlysulfoxide, all at 0.1 % v/v) that may potentially be present in the environment of the host *K. polythalamius* were also tested but no growth was detected even upon prolonged incubation.

Many nitrogen-fixing bacteria grow best under microaerophilic conditions, at least in part because nitrogenases are irreversibly inactivated by oxygen. We tested the aerotolerance of strain 2141T on soft-agar STBA medium (0.2 % w/v Bacto agar) with or without combined nitrogen source (ammonium chloride). In the presence of combined nitrogen, growth was observed from the surface to the bottom of the agar stab, while in the absence of combined nitrogen, growth was limited to subsurface regions of the agar, signifying a preference for microaerobic conditions during diazotrophic growth. The capacity of these symbionts to utilize nitrate and nitrite (5 mM final concentration on STBA plates) were tested during growth under thioautotrophic conditions by replacing ammonium chloride in STB50 medium. Under aerobic conditions, large colonies formed on nitrate plates, while small colonies developed on nitrite plates, suggesting that strain 2141T can assimilate nitrate and to a lesser extent, nitrite. Chemolithoautotrophic growth was also seen on both nitrate and nitrite plates under anaerobic conditions (GasPak Anaerobic Pouch). Yeast extract (0.1 % w/v) also supported growth under oxic growth conditions.

The effects of temperature, salt concentration and pH on cultures grown on STBA were determined under thioautotrophic conditions. Growth of strain 2141T was observed between 30 and 37 °C, with the optimum around 34 °C, while no growth was observed at 25 °C and 40 °C. Salt tolerance was tested by varying the concentration of NaCl from 0.0 M to 1.0 M, at 0.1 M increments in the liquid culture. The optimum salinity was 0.2 M NaCl, but growth was observed in NaCl concentrations ranging from 0.0 M to 0.7 M. Growth of strain 2141T was observed in media with an initial pH of 5.5 to 8.5, with pH 8.5 being the optimum. The pH buffer systems used were as follows: pH 5.5, 6.0 and 6.5 with 20 mM MES; pH 7.0, 7.5 and 8.0 with 20 mM HEPES; pH 8.5 with 20 mM TAPS; and pH 9.0 and 9.5 with 20 mM CHES. Three days after growth was initiated at pH 8.5, a dramatic decline in pH (>3 pH units) was observed, presumably due to the build-up of sulfate, the end-product of sulfur metabolism in thioautotrophic bacteria. Typical of marine bacteria, growth of strain 2141T requires concentrations of Ca^+2^ and Mg^+2^ ions similar to those found in seawater. No growth was seen on STBA medium modified to contain reduced Ca^+2^ and Mg^+2^ concentrations (0.5 mM CaCl_2_·2H_2_O and 0.05 mM MgCl_2_·6H_2_O); while growth was observed with 10 mM CaCl_2_·2H_2_O and 50 mM MgCl_2_·6H_2_O.

## Description of *Thiosocius* gen. nov.

*Thiosocius* (Thi.o.so′ci.us. Gr. n. *theion* sulfur; L. masc. n. *socius* partner; N.L. masc. n. *Thiosocius* a sulfur partner).

The monospecific genus *Thiosocius* is proposed to accommodate six thioautotrophic endosymbiotic bacterial strains isolated from the gill tissues of the giant shipworm *Kuphus polythalamius*, collected in two locations in the Philippines. All strains share greater than 99.8 % nucleotide identity within a 1265 bp region of the small subunit (16S) ribosomal RNA gene.

Gram-negative straight rods, that are on an average 1.7 µm long and 0.5 µm wide, have a single polar flagellum. Cells display carboxysomes and sulfur storage globules within the cytoplasm. Capable of chemolithoautotrophic growth utilizing thiosulfate as an energy source, and bicarbonate as a sole carbon source, diazotrophic, mesophilic, requires media containing elevated Ca and Mg concentrations reflecting those characteristic of seawater, capable of heterotrophic growth utilizing organic acids or alcohols, aerobic, microaerobic or facultative anaerobe via nitrate or nitrite respiration. The type species is *Thiosocius teredinicola*.

## Description of *Thiosocius teredinicola* sp. nov.

*Thiosocius teredinicola* [te.re.di.ni′co.la. L. n. *teredo* shipworm; L. suff.- *cola* (from L. n. *incola*), dweller; N.L. n. *teredinicola* shipworm-dweller].

Gram-negative rods, that are on an average 1.7 µm long and 0.5 µm wide, have a single polar flagellum, bearing carboxysomes and sulfur storage globules. Capable of chemolithoautotrophic growth on sulfide, thiosulfate, tetrathionate, or elemental sulfur on a completely chemically defined marine salt medium. Diazotrophic when grown in the absence of combined nitrogen. Assimilates nitrate and nitrite during aerobic thioautotrophic growth on thiosulfate. Can respire nitrate and nitrite anaerobically during thioautotrophic growth on thiosulfate. Grows heterotrophically on acetate, propionate, pyruvate, malate, succinate, fumarate, glycerol, ethanol, d-fructose, glutamine and glutamate. The FAME profile was largely composed of C_16 : 1_ ω7*c* and/or C_16 : 1_ ω6*c*, palmitic acid (C_16 : 0_) and (C_18 : 1_ ω7*c*). The pH, temperature and salinity optima for growth were found to be 8.5, 34 °C and 0.2 M NaCl, respectively. The closed-circular genome has been sequenced and has a G+C content of 60.1 % and is 4.79 Mbp in size. The type strain PMS-2141T.STBD.0c.01a^T^ (=DSM 108030^T^) was isolated from a sediment-dwelling *Kuphus polythalamius* from specimen ID PMS-2133X collected in a marine bay in the Philippines.

## Supplementary Data

Supplementary File 1

## References

[1] Dubilier N, Bergin C, Lott C (2008). Symbiotic diversity in marine animals: the art of harnessing chemosynthesis. Nat Rev Microbiol.

[2] Cavanaugh CM, McKiness ZP, Newton IL, Stewart FJ (2006). Marine chemosynthetic symbioses. The Prokaryotes.

[3] Huber M (2015). Compendium of Bivalves 2. A Full-Color Guide to the Remaining Seven Families. A Systematic Listing of 8,500 Bivalve Species and 10,500 Synonyms.

[4] Distel DL, Amin M, Burgoyne A, Linton E, Mamangkey G (2011). Molecular phylogeny of Pholadoidea Lamarck, 1809 supports a single origin for xylotrophy (wood feeding) and xylotrophic bacterial endosymbiosis in Bivalvia. Mol Phylogenet Evol.

[5] Waterbury JB, Calloway CB, Turner RD (1983). A cellulolytic nitrogen-fixing bacterium cultured from the gland of deshayes in shipworms (bivalvia: teredinidae). Science.

[6] Distel DL, Morrill W, Maclaren-Toussaint N, Franks D, Waterbury J (2002). *Teredinibacter turnerae* gen. nov., sp. nov., a dinitrogen-fixing, cellulolytic, endosymbiotic gamma-proteobacterium isolated from the gills of wood-boring molluscs (Bivalvia: Teredinidae). Int J Syst Evol Microbiol.

[7] O'Connor RM, Fung JM, Sharp KH, Benner JS, McClung C (2014). Gill bacteria enable a novel digestive strategy in a wood-feeding mollusk. Proc Natl Acad Sci USA.

[8] Shipway JR, Altamia MA, Haga T, Velásquez M, Albano J (2018). Observations on the Life History and Geographic Range of the Giant Chemosymbiotic Shipworm *Kuphus polythalamius* (Bivalvia: Teredinidae). The Biological Bulletin.

[9] Distel DL, Altamia MA, Lin Z, Shipway JR, Han A (2017). Discovery of chemoautotrophic symbiosis in the giant shipworm *Kuphus polythalamia* (Bivalvia: Teredinidae) extends wooden-steps theory. Proc Natl Acad Sci USA.

[10] Altekar G, Dwarkadas S, Huelsenbeck JP, Ronquist F (2004). Parallel Metropolis coupled Markov chain Monte Carlo for Bayesian phylogenetic inference. Bioinformatics.

[11] Rinke C, Schmitz-Esser S, Stoecker K, Nussbaumer AD, Molnár DA (2006). *"Candidatus* Thiobios zoothamnicoli," an ectosymbiotic bacterium covering the giant marine ciliate *Zoothamnium niveum*. Appl Environ Microbiol.

[12] Nakagawa S, Shimamura S, Takaki Y, Suzuki Y, Murakami S (2014). Allying with armored snails: the complete genome of gammaproteobacterial endosymbiont. Isme J.

[13] Suzuki Y, Kojima S, Sasaki T, Suzuki M, Utsumi T (2006). Host-symbiont relationships in hydrothermal vent gastropods of the genus *Alviniconcha* from the Southwest Pacific. Appl Environ Microbiol.

[14] Kuever J, Sievert SM, Stevens H, Brinkhoff T, Muyzer G (2002). Microorganisms of the oxidative and reductive part of the sulphur cycle at a shallow-water hydrothermal vent in the Aegean Sea (Milos, Greece). Cah Biol Mar.

[15] Roeselers G, Newton IL, Woyke T, Auchtung TA, Dilly GF (2010). Complete genome sequence of *Candidatus* Ruthia magnifica. Stand Genomic Sci.

[16] Kuwahara H, Yoshida T, Takaki Y, Shimamura S, Nishi S (2007). Reduced genome of the thioautotrophic intracellular symbiont in a deep-sea clam, *Calyptogena okutanii*. Curr Biol.

[17] Nussbaumer AD, Fisher CR, Bright M (2006). Horizontal endosymbiont transmission in hydrothermal vent tubeworms. Nature.

[18] Harmer TL, Rotjan RD, Nussbaumer AD, Bright M, Ng AW (2008). Free-living tube worm endosymbionts found at deep-sea vents. Appl Environ Microbiol.

[19] Won YJ, Hallam SJ, O'Mullan GD, Pan IL, Buck KR (2003). Environmental acquisition of thiotrophic endosymbionts by deep-sea mussels of the genus bathymodiolus. Appl Environ Microbiol.

[20] Flood BE, Jones DS, Bailey JV (2015). *Sedimenticola thiotaurini* sp. nov., a sulfur-oxidizing bacterium isolated from salt marsh sediments, and emended descriptions of the genus *Sedimenticola* and *Sedimenticola* selenatireducens. Int J Syst Evol Microbiol.

[21] Goris J, Konstantinidis KT, Klappenbach JA, Coenye T, Vandamme P (2007). DNA-DNA hybridization values and their relationship to whole-genome sequence similarities. Int J Syst Evol Microbiol.

[22] Kim M, Oh HS, Park SC, Chun J (2014). Towards a taxonomic coherence between average nucleotide identity and 16S rRNA gene sequence similarity for species demarcation of prokaryotes. Int J Syst Evol Microbiol.

[23] Qin QL, Xie BB, Zhang XY, Chen XL, Zhou BC (2014). A proposed genus boundary for the prokaryotes based on genomic insights. J Bacteriol.

[24] Abdul-Rahman F, Petit E, Blanchard JL (2013). The distribution of polyhedral bacterial microcompartments suggests frequent horizontal transfer and operon reassembly. J Phylogenetics Evol Biol.

[25] Polz MF, Felbeck H, Novak R, Nebelsick M, Ott JA (1992). Chemoautotrophic, sulfur-oxidizing symbiotic bacteria on marine nematodes: Morphological and biochemical characterization. Microb Ecol.

[26] Krueger DM, Dubilier N, Cavanaugh CM (1996). Chemoautotrophic symbiosis in the tropical *clamSolemya occidentalis* (Bivalvia: Protobranchia): ultrastructural and phylogenetic analysis. Mar Biol.

[27] Bauer-Nebelsick M, Bardele CF, Ott JA (1996). Electron microscopic studies on *Zoothamnium niveum* (Hemprich & Ehrenberg, 1831) Ehrenberg 1838 (Oligohymenophora, Peritrichida), a ciliate with ectosymbiotic, chemoautotrophic bacteria. Eur J Protistol.

[28] Lechene CP, Luyten Y, McMahon G, Distel DL (2007). Quantitative imaging of nitrogen fixation by individual bacteria within animal cells. Science.

[29] König S, Gros O, Heiden SE, Hinzke T, Thürmer A (2016). Nitrogen fixation in a chemoautotrophic lucinid symbiosis. Nat Microbiol.

[30] Petersen JM, Kemper A, Gruber-Vodicka H, Cardini U, van der Geest M (2016). Chemosynthetic symbionts of marine invertebrate animals are capable of nitrogen fixation. Nat Microbiol.

[31] Kleiner M, Wentrup C, Lott C, Teeling H, Wetzel S (2012). Metaproteomics of a gutless marine worm and its symbiotic microbial community reveal unusual pathways for carbon and energy use. Proc Natl Acad Sci USA.

