# Supplementary material for: Thiosocius teredinicola gen. nov., sp. nov., a sulfur-oxidizing chemolithoautotrophic endosymbiont cultivated from the gills of the giant shipworm, Kuphus polythalamius
Source: Int J Syst Evol Microbiol. 2018 Dec 12;69(3):638–44. doi: 10.1099/ijsem.0.003143 (PMC7705117; doi:10.1099/ijsem.0.003143)
Supplement: Supplementary File 1 [file ijsem-69-638-s001.pdf]

Supplemental Figures and Tables for:

Title: *Thiosocius teredinicola* gen. nov., sp. nov., a sulfur-oxidizing chemolithoautotrophic endosymbiont cultivated from the gills of the giant shipworm, *Kuphus polythalamius*

Journal: International Journal of Systematic and Evolutionary Microbiology (IJSEM)

Authors: Marvin A. Altamia[1], J. Reuben Shipway[2], Gisela P. Concepcion[1], Margo G. Haygood [3*] and Daniel L. Distel [2*]

Author affiliations: [1]Marine Science Institute, University of the Philippines, Diliman, Quezon City, Philippines; [2] Department of Marine and Environmental Science, Ocean Genome Legacy Center, Northeastern University, 430 Nahant Road, Nahant MA 01908, USA; [3] Department of Medicinal Chemistry, University of Utah, Salt Lake City, UT 84112, USA

*Correspondence: Daniel L. Distel, d.distel@neu.edu, Margo G. Haygood, margo.haygood@utah.edu

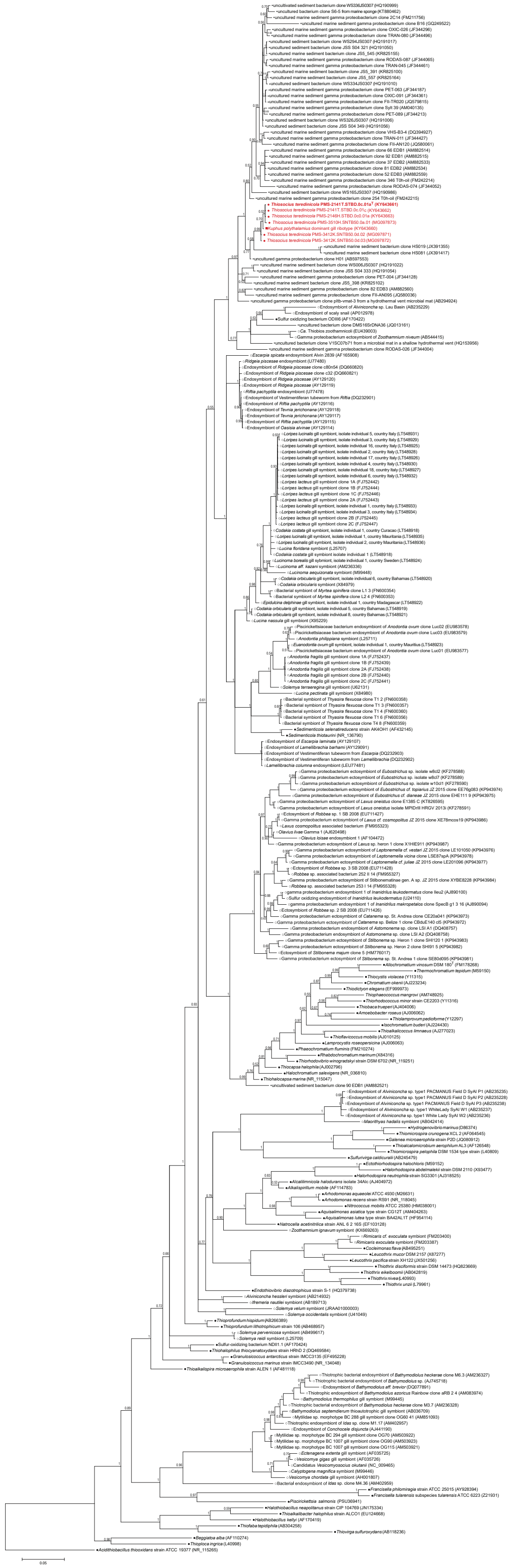

Figure S1: Phylogenetic relationships of *Thiosocius teredinicola* strains and other related free-living and symbiotic bacteria based on 16S rRNA gene sequences. A Bayesian tree was constructed using 1,172 nucleotide positions employing GTR + I + Γ as the substitution model. Chain length was set to 4 million, subsampling every 2,000 generations and discarding the first 20% of the analytical results as burn-in. Posterior probability values are indicated for each node. The scale bar represents nucleotide substitution rate per site. Closed circles, bacterial isolates; open circles, uncultivated symbionts; asterisks, environmental clones; closed square, sequence recovered from *K. polythalamius* gill metagenome. Isolates described in this study appear highlighted in red. Figure 1 in the main text is a subtree excerpted from the tree presented here. For best viewing, zoom electronically 400-800%.

(A)

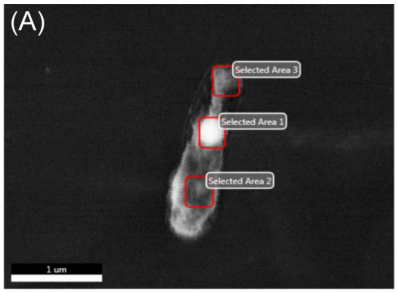

(B) Selected Area 1

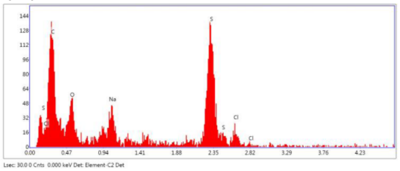

(C)

Element	Weight %	Atomic %	Net Int.	Error %
C K	42.91	65.17	27.22	16.24
O K	3.41	3.89	4.41	46.08
NaK	2.83	2.25	4.46	36.80
S K	46.60	26.51	41.11	9.84
ClK	4.25	2.19	2.64	57.65

(D) Selected Area 2

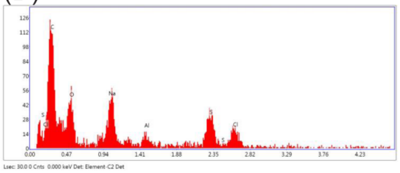

(E)

Element	Weight %	Atomic %	Net Int.	Error %
C K	53.88	72.49	23.53	16.74
O K	6.08	6.14	3.92	57.12
NaK	8.84	6.21	6.76	26.71
AlK	0.15	0.09	0.12	99.99
S K	19.01	9.58	8.18	17.82
ClK	12.04	5.49	3.75	34.91

(F) Selected Area 3

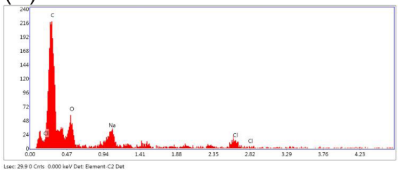

(G)

Element	Weight %	Atomic %	Net Int.	Error %
C K	71.89	82.22	54.12	10.35
O K	11.99	10.29	7.18	23.89
NaK	5.91	3.53	4.08	37.21
ClK	10.21	3.95	2.99	36.03

Figure S2. Energy dispersive X-ray (EDX) analysis to determine the elemental composition of putative sulfur-globules. (A) A single cell of strain 2141T grown on STB50 chemolithoautotrophic liquid culture medium. Three regions of interest (selected areas 1-3) were chosen for EDX analysis: selected areas 1and 2 containing large and small putative sulfur globules respectively; and selected area 3, corresponding to the putative bacterial cytoplasm. (B,D,F) EDX spectra and (C,E,G) tabular data corresponding to the elemental composition of selected areas 1-3, respectively. Note, sulfur represents 46.6% and 19% of the total elemental composition within the selected areas containing putative large and small sulfur-globules respectively but is not detectable in the putative bacterial cytoplasm. Scale bar = 1 µm.

**Table S1.** Fatty acid compositions of polar lipids in *Thiosocius teredinicola* strain 2141T^{T}.	
**Fatty acid**	**Percentage**
$C_{10:0}$	0.5
$C_{10:0}$ 3OH	2.8
$C_{12:0}$	0.4
$C_{12:1}$ 3OH	0.5
$C_{12:0}$ 3OH	0.4
$C_{14:0}$	0.6
$C_{15:0}$ anteiso	0.3
$C_{15:0}$	0.0
summed feature 2 ($C_{14:0}$ 3OH / $C_{16:1}$ iso I)	1.6
$C_{16:0}$ iso	0.2
summed feature 3 ($C_{16:1}$ ω7*c* / $C_{16:1}$ ω6*c*)	40.1
$C_{16:0}$	33.4
$C_{17:1}$ ω8*c*	0.5
$C_{17:1}$ ω6*c*	0.4
$C_{17:0}$	1.6
$C_{16:0}$ 3OH	0.4
$C_{18:1}$ ω9*c*	0.6
summed feature 8 ($C_{18:1}$ ω7*c*)	11.9
$C_{18:1}$ ω5*c*	0.3
$C_{18:0}$	3.5
$C_{20:1}$ ω7*c*	0.2
